# Clinical effects of anweiyang capsule and *Pinellia* decoction for eradication *of Helicobacter pylori* and healing of peptic ulcers

**DOI:** 10.1186/s13568-024-01783-4

**Published:** 2024-11-08

**Authors:** Xiaoying Feng, Xin Zhao, Lanlan Cao, Ruixue Shang, Xinran Feng

**Affiliations:** 1 Department of Traditional Chinese Medicine, Xingtai Third Hospital, Xingtai, 054001 Hebei Province China; 2 Beijing Tiantongyuan North Community Health Service Center, Changping District, Beijing, 102200 China; 3 Xingtai Third Hospital, Xingtai, 054001 Hebei Province China

**Keywords:** Anweiyang capsule, *Pinellia* heart-draining decoction, Peptic ulcer, *Helicobacter pylori*

## Abstract

Peptic ulcers are a prevalent and often chronic condition within the digestive system, frequently prone to recurrence. This study aims to evaluate the clinical effects of Anweiyang capsule combined with *Pinellia* Heart-Draining Decoction on *Helicobacter pylori* eradication, ulcer healing rates, and improvement of Traditional Chinese Medicine (TCM) syndromes in patients with peptic ulcers. A total of 100 patients were randomly assigned to an observation group (*n* = 50), receiving the herbal combination, and a control group (*n* = 50), receiving standard Western medical treatment. The total effective rate was significantly (*P* < 0.05) higher in the observation group (96%) compared to the control group (80%). After 4 weeks of treatment, serum procalcitonin (PCT) and C-reactive protein (CRP) levels decreased, while prostaglandin E2 (PGE2) levels increased in both groups; however, the observation group exhibited better results (*P* < 0.05). The observation group achieved a *H. pylori* eradication rate of 94% and an ulcer healing rate of 82%, compared to 64% and 56% in the control group, respectively. Improvement in TCM syndrome scores was more significant in the observation group (*P* < 0.05). Moreover, the incidence of adverse reactions was significantly (*P* < 0.05) lower in the observation group (4%) compared to the control group (18%). In conclusion, Anweiyang capsule combined with *Pinellia* Heart-Draining Decoction significantly enhances the eradication rate of *H. pylori* and the ulcer healing rate without substantially increasing the risk of adverse reactions, demonstrating its safety and reliability for patients.

## Introduction

Peptic ulcer is an inflammatory lesion in the gastrointestinal mucosa, primarily caused by the corrosive effects of gastric acid and digestive enzymes. This condition can breach the mucosal muscle layer or penetrate deeper tissues (Kavitt et al. 2019). Peptic ulcers typically occur in the stomach and duodenum, but can also develop near esophagogastric anastomoses, gastrojejunal anastomoses, or in Meckel diverticulum with gastric mucosa (Kamada et al. 2021). Estimates suggest that around 10% of the population will experience a peptic ulcer at some point in their lives. Symptoms include upper abdominal pain, nausea, vomiting, and indigestion, and recurrent episodes can significantly impact a patient’s daily life and work (Dunlap and Patterson 2019). Thus, symptomatic treatment is essential for alleviating discomfort.

Research has established that *Helicobacter pylori* infection is a leading cause of peptic ulcers (Clarke et al. 2022). The causes of *H. pylori* infection may include contact with the saliva, vomit, or fecal matter of an infected person, as well as poor hygiene (Stefano et al. 2018). Eradicating *H. pylori* is crucial for effective ulcer management (Joo et al. 2020). Western medicine primarily employs a quadruple therapy consisting of secretory agents and proton pump inhibitors—specifically, esomeprazole, amoxicillin, clarithromycin, and colloidal bismuth. This combination effectively inhibits *H. pylori* proliferation, reduces gastric mucosal damage, and alleviates gastrointestinal symptoms (Ardalani et al. 2020). However, *H. pylori* resistance can lower eradication rates, compromise the quality of ulcer healing, and lead to significant side effects, including persistent gastritis, peptic ulcers, gastroesophageal reflux disease, anemia, and an increased risk of gastric cancer (Bauer and Meyer 2011). Additionally, it can cause relapses, imposing economic burdens and unnecessary suffering on patients. (Almadi et al. 2024a, b). Therefore, finding more effective methods to enhance *H. pylori* clearance is imperative.

In traditional Chinese medicine (TCM), peptic ulcers are classified under “stomachache” and “gastric hospital carbuncle” (Ahn 2020). Factors such as improper diet, external environmental influences, and emotional stress can damage the spleen and stomach, resulting in symptoms like qi stagnation, phlegm obstruction, abdominal distension, belching, and weakness, often accompanied by a pale, fatty tongue and a weak pulse (Quah et al. 2019; Shirazi et al. 2020). Treatment should focus on tonifying the spleen and stomach while regulating qi flow. TCM has a long-standing tradition of treating peptic ulcers through syndrome differentiation and therapeutic adjustments, accumulating valuable clinical experience over time (Jia et al. 2023).

Anweiyang capsule, a proprietary Chinese medicine preparation derived from *Glycyrrhiza glabra* flavonoids, is known for its consolidating effects. It effectively inhibits stomach acid production, regulates metabolic functions, and minimizes adverse reactions (Joo 2020; Assefa et al. 2022). Additionally, *Pinellia* Heart-Draining Decoction has been shown to repair microvascular networks, increase gastric mucosal growth factors, enhance gastrointestinal hormone levels, regulate gastric mucosal micro-perfusion, and protect the gastric lining (Serafim et al. 2020; Saleh et al. 2021; Okimoto et al. 2021; Moeller et al. 1962). Recently, a combination of traditional Chinese and Western medicine has yielded promising results in treating peptic ulcers (Sbeit et al. 2021). However, further research is needed to draw more scientifically rigorous conclusions. This study investigates the efficacy and safety of Anweiyang Capsule combined with *Pinellia* Heart-Draining Decoction alongside Western medicine quadruple therapy on *H. pylori* eradication, ulcer healing rates, and improvement of Traditional Chinese Medicine (TCM) syndromes, aiming to provide clinicians with effective treatment options and objective evaluation criteria.

## Materials and methods

### Study ethics

Informed written consent was obtained from the patients, along with consent from family members who were willing to provide assistance in the study. Approval for this study was obtained from our hospital’s The Medical Ethics Committee of Xingtai Third Hospital, Xingtai, Hebei, China (Reference number: XTH79901).

### Patient sampling and group allocation

From September 2020 to September 2022, a total of 100 patients with peptic ulcers treated at Xingtai Third Hospital, Xingtai, Hebei, China were randomly assigned to two groups: the observation group (*n* = 50) and the control group (*n* = 50). The control group comprised 25 men and 25 women, with ages ranging from 48 to 73 years (mean age = 54.93 ± 3.09). Their weights varied from 56 to 87 kg (mean weight = 65.41 ± 9.32), and the duration of the disease ranged from 2 to 4 years (mean = 3.29 ± 0.23). Disease subtypes included 17 cases of gastric ulcer, 24 cases of duodenal ulcer, and 9 cases of pyloric canal ulcer.

In the observation group, there were 27 men and 23 women, aged 47 to 70 years (mean age = 54.84 ± 3.11). Their weights ranged from 55 to 88 kg (mean weight = 65.62 ± 9.51), with a disease duration of 2 to 4 years (mean = 3.31 ± 0.19). The distribution of disease types was as follows: gastric ulcer (*n* = 19), duodenal ulcer (*n* = 23), and pyloric ulcer (*n* = 8). Regarding education level, 22 patients had completed senior high school or technical secondary school, and 16 had a junior college education or higher. No significant differences were observed in general characteristics between the groups (*P* > 0.05).

Sample size (*n*_1_) was calculated by this formula,

$$\:{n}_{1}=\frac{{[{\text{Z}}_{{\upalpha\:}/2}\sqrt{p\left(1-p\right)\left(1+\text{c}\right)/\text{c}}\:+{\text{Z}}_{{\upbeta\:}}\sqrt{{p}_{1}(1-{p}_{1})\:+\:{p}_{2}\:(1-{p}_{2})/\text{c}]}}^{2}}{{\left({p}_{1}-{p}_{2}\right)}^{2}}$$

The bilateral α is taken as 0.05 and β as 0.20. The therapeutic effect (total effective rate) is considered the effect index, with parameters set as *p*_*1*_ = 0.96, and *p*_2_ = 0.77. Based on the calculations, each group requires a sample size of 45 cases, with a 10% anticipated drop-off rate. Therefore, each group includes about 50 patients, resulting in a total of 100 patients.

### Diagnostic criteria

The diagnostic criteria for peptic ulcer include both Western and Traditional Chinese Medicine (TCM) approaches. Western medicine practitioners refer to the “Evaluation and Diagnostic Criteria of *H. pylori* Infection” (Godbole et al. 2020) and “Practical Internal Medicine” (Chen 2005) for guidance. In TCM, clinicians base their criteria on the guiding principles for clinical research of new drugs (Zhang and Zhang 2021). Key symptoms include epigastric burning pain, characterized by warmth and pressure, along with secondary symptoms such as dry mouth or sour regurgitation, noisy gastric sounds, belching, cold extremities, dry stools, a pale or light red tongue, a thick tongue with tooth marks, a yellow-white or greasy coating, and a fine pulse.

### Inclusion criteria

Participants in the study had to meet several inclusion criteria. First, all cases had to have a positive *H. pylori* test result that aligned with the diagnostic criteria of both Traditional Chinese Medicine and Western medicine. Second, patients had to be at least 18 years old. Third, fiberoptic endoscopy had to confirm that participants were in the active stage of ulcer within one week of enrollment, with the ulcer diameter ranging from 3 mm to 20 mm. Finally, all patients had to sign an informed consent form to participate in the study.

### Exclusion criteria

The study excluded individuals who met any of the following conditions: patients with gastric ulcers caused by other etiologies, those with hematological disorders, patients with severe infections, individuals who had a history of gastrointestinal surgery, and those with a history of alcohol or drug abuse. Additionally, patients with severe organic diseases affecting the heart, liver, kidneys, or other organs were excluded, as were individuals with a history of cancer or severe mental disorders. Pregnant or lactating women, individuals with known allergies to the study drugs, and those who had received anti-*Helicobacter pylori* treatment in the past three months were also excluded from participation.

### Treatment scheme

**Fig. 1:**
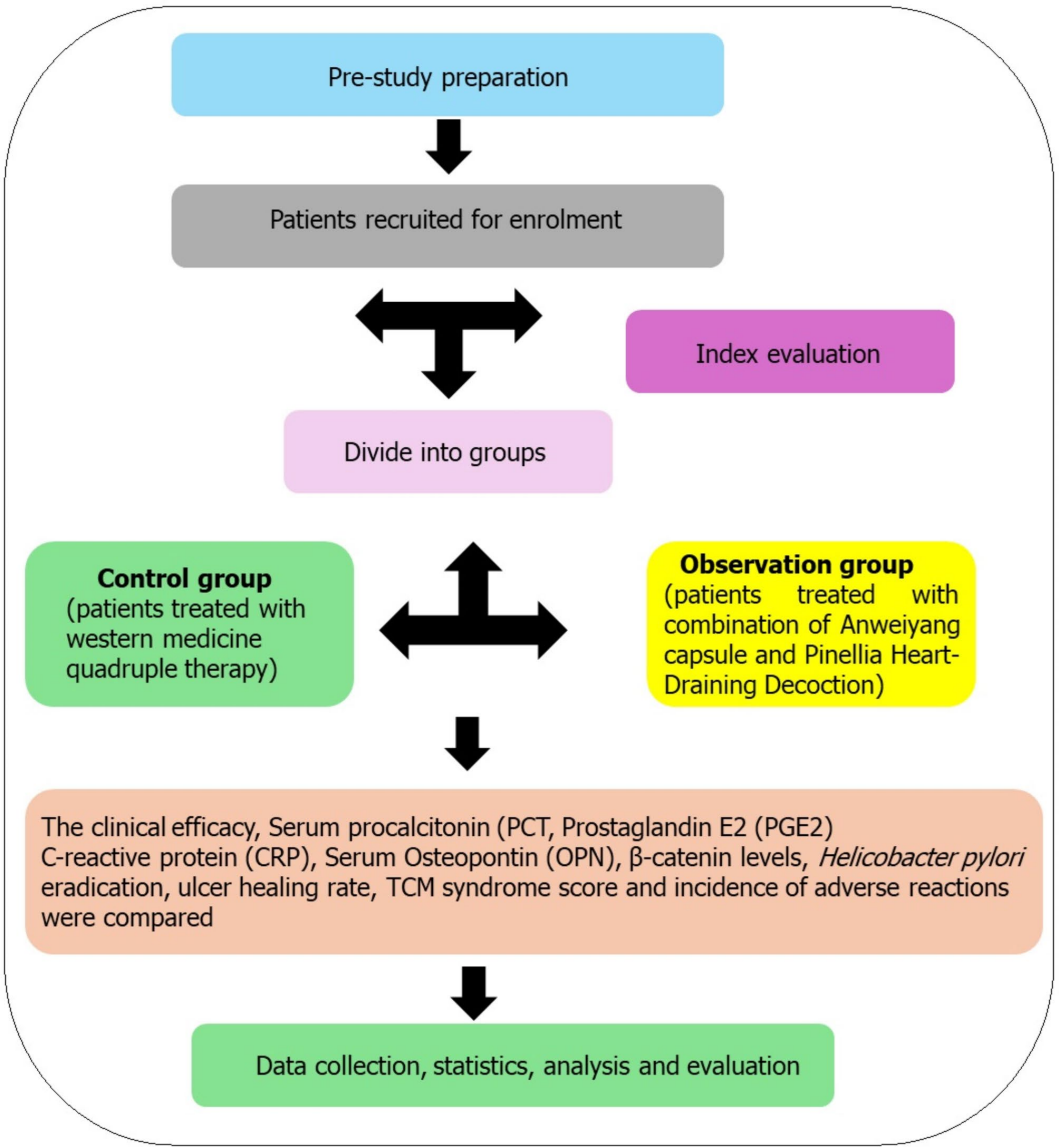
Illustration of study design

The treatment scheme, illustrated in Fig. 1, was implemented. Patients in the control group received a combination therapy consisting of esomeprazole (AstraZeneca Pharmaceutical Co., Ltd., Sinopharm Zhunzi H20046379), amoxicillin (Yuekang Pharmaceutical Group Co., Ltd., Sinopharm Zhunzi H11020396), clarithromycin (Sinopharm Shantou Jinshi Pharmaceutical Co., Ltd., Sinopharm Zhunzi H1991164), and colloidal bismuth pectin (Zhejiang Deende Pharmaceutical Co., Ltd., Sinopharm Zhunzi H200644). The dosages were as follows: esomeprazole at 20 mg, orally, twice daily; amoxicillin at 1 g, orally, twice daily; clarithromycin at 0.5 g, orally, twice daily; and colloidal bismuth pectin at 150 mg, orally, three times daily. Treatment for the control group lasted 4 weeks. In the observation group, patients received Anweiyang capsules and *Pinellia* Heart-Draining Decoction in addition to the same regimen as the control group. Anweiyang capsules (Xinjiang Quanan Pharmaceutical Co., Ltd., Chinese medicine Z10950062) were administered at a dosage of 0.4 g, orally, three times daily. The *Pinellia* Heart-Draining Decoction included 12 g of *Pinellia ternata*, 15 g of *Codonopsis pilosula*, 10 g of tangerine (*Citrus tangerine*) peel, 10 g of *Scutellaria baicalensis*, 6 g of *Glycyrrhiza glabra*, 6 g of *Coptis chinensis*, and 6 g of dried ginger (*Zingiber officinale*), prepared in 11 doses per day, with the decoction taken in the morning and evening. Additional ingredients could be added based on specific symptoms, such as *Salvia* for stomach ligament stagnation, gypsum for excessive heat, *Hawthorn* for gastrointestinal stagnation, *Yam* and aromatic herb for cold invasion, *Heshouwu* for stomach yin deficiency, *White Peony* and *Bupleurum* for liver and stomach qi stagnation, and *Dang Sheng* for spleen and stomach cold deficiency. The decoction was to be taken 30–60 min before meals, and the treatment duration for the observation group was also 4 weeks.

### Observation indices

#### Clinical effect

The diagnosis and treatment of peptic ulcer using integrated traditional Chinese and Western medicine followed established standards (Huang et al. 2015). Clinical efficacy was categorized into four groups: cured (complete disappearance of symptoms, endoscopic healing, and eradication of *H. pylori*), remarkably effective (symptoms resolved with minimal inflammation remaining and eradication of *H. pylori*), effective (symptoms reduced by at least 50% with possible *H. pylori* status), and ineffective (persistent or worsening symptoms with positive *H. pylori*).

The total effective rate was calculated as,

$$ \frac{{Number\:of\:cured + Remarkably\:effective + Effective\:cases}}{{Total\:cases}} \times 100 $$

#### Serum levels of PCT, PGE2, and CRP

Blood samples were collected before and after treatment, and serum levels of procalcitonin (PCT), prostaglandin E2 (PGE2), and C-reactive protein (CRP) were measured using enzyme-linked immunosorbent assay (ELISA) after high-speed centrifugation (3,000 r/min for 10 min, Changsha Xiangrui Centrifuge Co., Ltd., model: TG16-WS).

#### Serum OPN and β-Catenin levels

Serum levels of osteopontin (OPN) and β-catenin were also measured pre- and post-treatment using ELISA after high-speed centrifugation (3,000 r/min for 10 min, Changsha Xiangrui Centrifuge Co., Ltd., model: TG16-WS).

#### *Helicobacter pylori* eradication and ulcer healing rates

The *H. pylori* eradication rate was assessed one-month post-treatment using the *H. pylori* urease test (Uotani and Graham 2015) with a negative result indicating successful eradication. The rate was calculated as

$$ \begin{gathered} H.\:pyroli\:eradication\:rate\: \hfill \\ \quad = \frac{{Number\:of\:H.\:pyroli\:eradication\:cases}}{{Total\:number\:of\:cases}}\: \times \:\:100 \hfill \\ \end{gathered} $$

Ulcer healing was defined by the complete disappearance of clinical symptoms and endoscopic evidence of healing or scarring (Arakawa et al. 2012).

#### Traditional Chinese medicine syndrome integral value

Patients were evaluated for TCM syndrome severity before and after treatment using quantitative grading criteria. Symptoms such as nausea, acid reflux, bloating, and stomach pain were scored on a scale of 0 (none) to 6 (severe), yielding a total score range of 0–24, with higher scores indicating more severe conditions.

#### Incidence of adverse reactions

Adverse reactions, including gastrointestinal, allergic reactions, and headaches, were recorded during the treatment period. The total incidence was calculated as,

$$\:\frac{Total\:number\:of\:adverse\:reactions\:}{Total\:number\:of\:cases}\:\times\:\:100$$

Gastrointestinal reactions included symptoms like stomachache, distension, nausea, vomiting, retching, and acid regurgitation. Allergic reactions were defined as immune responses causing tissue damage upon re-exposure to an antigen, while headaches were characterized by pain located from the eyebrow arch to the lower occipital and upper neck.

### Statistical analysis

The data were analyzed using SPSS version 21.0 statistical software. For measurements with a normal or approximately normal distribution, values are presented as means ± standard deviation (SD). Paired t-tests were used for within groups, while independent sample t-tests were employed for comparisons between groups. Categorical data are expressed as *n* (%), and the chi-square (χ²) test was utilized for these comparisons. A *p*-value of less than 0.05 was considered statistically significant.

## Results

### Comparison of therapeutic effects

The observation group demonstrated a total effective rate of 96%, with 41 patients cured, 5 showing remarkable improvement, 2 classified as effective, and 2 as ineffective. In contrast, the control group had 28 patients cured, 4 with remarkable improvement, 8 effective, and 10 ineffective, resulting in a total effective rate of 80.00% (*P* < 0.05) (Table 1).

**Table 1:** Comparison of therapeutic effects between Observation and Control Groups

Group	*n*	Cured	Remarkable effect	Effective	Invalid	Total efficiency(%)
Observation group	50	41(82%)	5(10%)	2(4%)	2(4%)	48(96%)
Control group	50	28(56%)	4(8.00)	8(16%)	10(20%)	40(80%)
*χ2*						6.061
*P*						< 0.05

### Serum levels of PCT, PGE2, and CRP before and after treatment

Before treatment, there was no significant difference in serum levels of PCT, PGE2, and CRP between the groups (*P* > 0.05). After 4 weeks of treatment, both groups showed a decrease in serum PCT and CRP levels and an increase in serum PGE2 levels compared to baseline. Notably, the observation group demonstrated significantly (*P* < 0.05) improved serum levels of PCT, PGE2, and CRP compared to the control group (Table 2).

**Table 2:** Comparison of serum PCT, PGE2, and CRP levels pre- and Post-treatment in in Study groups

Group	Serum PCT (µg/L)		Serum PGE2 (ng/mL)		Serum CRP (mg/L)	
	Before treatment	After treatment	Before treatment	After treatment	Before treatment	After treatment
Observation group	2.76 ± 0.19	0.46 ± 0.15^a^	181.92 ± 15.18	211.32 ± 18.24^a^	11.67 ± 4.17	5.25 ± 0.19^a^
Control group	2.75 ± 0.18	1.26 ± 0.23^b^	181.93 ± 15.17	201.05 ± 15.21^b^	11.71 ± 4.16	8.97 ± 2.33^b^
*t*	0.270	5.150	0.003	3.057	0.048	11.252
*P*	> 0.05	< 0.05	> 0.05	< 0.05	> 0.05	< 0.05

Compared with the observation group before treatment, ^a^*P* < 0.05; compared with the control group before treatment, ^b^*P* < 0.05

### Serum osteopontin (OPN) and β-catenin levels before and after treatment

Prior to treatment, serum levels of Osteopontin (OPN) and β-catenin did not exhibit statistically significant differences between the observation and control groups (*P* > 0.05), indicating comparable baseline characteristics. After a treatment period of 4 weeks, the observation group demonstrated a significant decrease in serum OPN levels, measuring 51.03 ± 2.11 µg/L compared to the baseline level of 60.63 ± 5.45 µg/L (*P* < 0.05). Similarly, serum β-catenin levels in the observation group significantly decreased from 16.44 ± 2.45 µg/L to 12.13 ± 0.19 µg/L (*P* < 0.05). In contrast, the control group exhibited a reduction in OPN and β-catenin levels; however, these changes were not statistically significant when compared to baseline measurements (*P* > 0.05 for both) (Table 3).

**Table 3:** Serum OPN and β-catenin levels before and after treatment in study groups compared with the observation group before treatment, ^a^*P* < 0.05; compared with the control group before treatment, ^b^*P* < 0.05

Group	Serum OPN (µg/L)		Serum β-catenin (µg/L)	
	Before treatment	After treatment	Before treatment	After treatment
Observation group	60.63 ± 5.45	51.03 ± 2.11a	16.44 ± 2.45	12.13 ± 0.19a
Control group	60.58 ± 5.47	55.37 ± 3.04b	16.39 ± 2.41	14.41 ± 1.29b
*t*	0.046	8.293	0.103	12.364
*P*	>0.05	<0.05	>0.05	<0.05

### *Helicobacter pylori* eradication rate and ulcer healing rate

The results indicated significant differences in the eradication of *H. pylori* and ulcer healing between the observation and control groups. In the observation group, a total of 47 patients achieved *H. pylori* eradication, resulting in an eradication rate of 94%. Additionally, 41 patients demonstrated complete healing of their ulcers, yielding an ulcer healing rate of 82%. Conversely, in the control group, only 32 patients successfully eradicated *H. pylori*, leading to an eradication rate of 64%. The ulcer healing rate in this group was significantly lower, with only 28 patients healing, corresponding to a rate of 56%. The differences in *both H. pylori* eradication and ulcer healing rates between the two groups were statistically significant (*P* < 0.05) (Table 4).

**Table 4:** *H. Pylori* Eradication Rate and Ulcer Healing Rate in Study groups

Group	*n*	H. pylori Eradication rate	Ulcer healing rate
Observation group	50	47(94%)	41(82%)
Control group	50	32(64%)	28(56%)
*χ2*		13.562	7.901
*P*		< 0.05	< 0.05

### TCM syndrome scores before and after treatment

**Fig. 2:**
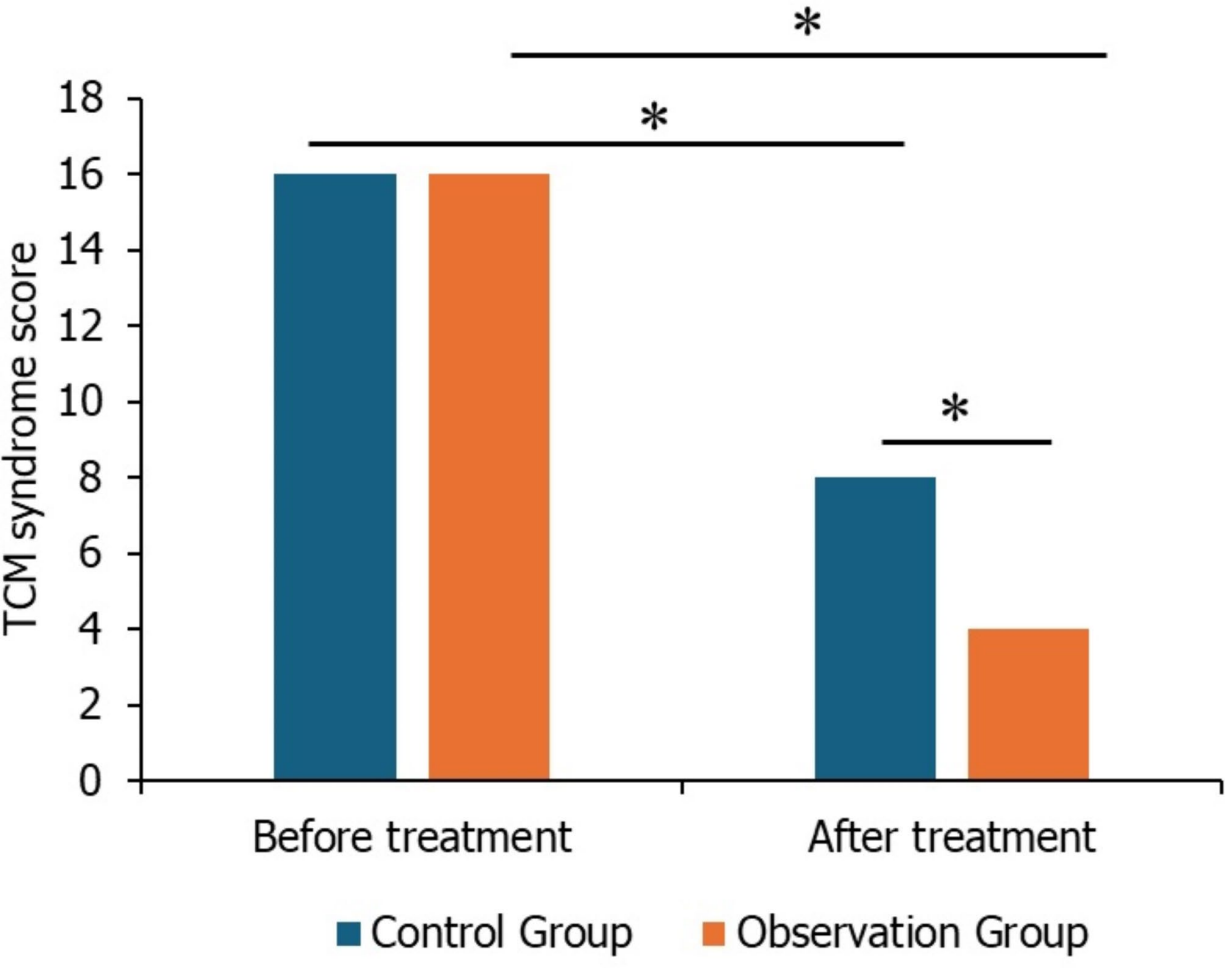
TCM Syndrome Scores Before and After Treatment in Study Groups (*P < 0.05)

Prior to treatment, there were no significant differences in TCM syndrome scores between the groups (*P* > 0.05). However, after 4 weeks of treatment, there was a significant reduction in TCM syndrome integral values, indicating improvement in symptoms. The observation group demonstrated significantly better scores compared to the control group (*P* < 0.05) (Fig. 2).

### The incidence of adverse reactions

In the study, the incidence of adverse reactions was evaluated in both groups. In the observation group of 50 patients, there were minimal adverse reactions, with only 1 patient experiencing a gastrointestinal reaction and 1 reporting a headache. This resulted in a total adverse reaction incidence of 4%. Conversely, the control group, also consisting of 50 patients, reported a higher incidence of adverse reactions. Specifically, 5 patients experienced gastrointestinal issues, 2 had allergic reactions, and 2 reported headaches, leading to a total incidence rate of 18%. (Table 5).

**Table 5:** Incidence of adverse reactions in study groups

Group	*N*	Gastrointestinal reaction	Allergic reaction	Headache	Total incidence rate
Observation group	50	1(2%)	0(0%)	1(2%)	2(4%)
Control group	50	5(10%)	2(4%)	2(4%)	9(18%)
χ2					5.005
*P*					< 0.05

## Discussion

The rising prevalence of peptic ulcers presents a significant public health concern, characterized by a protracted and recurrent course (Almadi et al. 2024a, b). Modern medicine attributes the development of peptic ulcers to factors such as the integrity of the gastric mucosal barrier and the regulation of gastric acid secretion (Yalcin et al. 2022). Furthermore, *Helicobacter pylori* infection plays a critical role in the onset, progression, and prognosis of peptic ulcers. Once *H. pylori* establish itself within the gastric mucosa, it disrupts local defence and repair mechanisms, promoting inflammation and increasing both gastric acid and gastrin secretion. This dual effect leads to mucosal damage and ulcer formation within the digestive tract (Yalcin et al. 2022).

Current Western medical practices primarily involve strategies that inhibit gastric acid secretion, protect the gastric mucosa, and eradicate *H. pylori* to foster an optimal healing environment (Tsoi et al. 2022). Standard treatment often includes a quadruple drug regimen, with esomeprazole rapidly suppressing acid secretion to create a low-acidity environment conducive for antibiotic efficacy. Amoxicillin and clarithromycin are commonly employed to target *H. pylori*, while colloidal bismuth pectin offers protective benefits to the gastrointestinal mucosa (Périco et al. 2020; Spósito et al. 2021; Rosen et al. 2021). Although quadruple therapy can alleviate symptoms during the acute phase, many patients experience recurrence, prolonged ulcer healing, or the emergence of chronic ulcers. Moreover, the extended use of these therapies heightens the risk of adverse effects, including drug allergies and damage to liver and kidney function. Thus, there is an urgent need for a safe, effective, and sustainable approach to prevent the progression of peptic ulcers while maximizing *H. pylori* eradication and promoting ulcer healing.

In recent years, Traditional Chinese Medicine (TCM) has garnered attention for its role in treating peptic ulcers associated with *H. pylori* infection. TCM classifies peptic ulcers under “epigastric pain” and “acid swallowing,” emphasizing the need to regulate the spleen and stomach, soothe the liver, and harmonize qi. *Pinellia* Heart-Draining Decoction, originating from the *Treatise on Febrile Diseases*, has demonstrated efficacy in addressing gastrointestinal and splenic dysfunction (Lai et al. 2020). Anweiyang Capsule is noted for its qi-tonifying and detoxifying properties, showing promise in managing peptic ulcers (Jiang et al. 2024).

The findings from our study revealed that the total effective rate in the observation group receiving the combined therapy was 96%, significantly higher than the control group’s 80%. The observation group achieved an *H. pylori* eradication rate of 94% and an ulcer healing rate of 82%. In contrast, the control group reported an eradication rate of 64% and a healing rate of 56%. After 4 weeks of treatment, the TCM syndrome integral was notably lower in the observation group, indicating that the combined therapy effectively alleviated clinical symptoms while enhancing *H. pylori* eradication and ulcer healing rates.

According to TCM principles, the stomach is regarded as the “sea of water and grain” and an essential organ for digestive health (Hao and Song 2023). Disruptions in dietary habits, as well as imbalances in cold and heat, can adversely affect gastric function, contributing to pathological changes. The primary symptom of peptic ulcers is epigastric pain, rooted in an imbalance between the spleen and stomach, often manifesting as “qi stagnation pain.” Additionally, this condition frequently correlates with acid reflux, which may stem from liver qi stagnation disrupting the harmony of stomach qi (Zaslona et al. 2020; Hao and Song 2023). Thus, effective treatment strategies should focus on acid inhibition, gastric protection, and the promotion of digestive health.

*Pinellia Heart-Draining Decoction* comprises several key ingredients, with *Pinellia ternata* as the principal herb, which alleviates nausea and vomiting while promoting the dispersion of stagnant energy. *Huang Lian* and *Scutellaria baicalensis* contribute to the formula’s heat-clearing and detoxifying properties. Complementary herbs such as *Chen Pi*, *Radix Codonopsis*, and *Dang Jiang* help tonify qi, strengthen the spleen, and alleviate coldness. Roasted licorice harmonizes the overall formulation (Lo et al. 2012). Together, these components work synergistically to invigorate the body while expelling pathogenic factors, regulating qi, and restoring stomach function. Anweiyang, a licorice (*Glycyrrhiza glabra*) extract primarily composed of flavonoids, also offers protective benefits to the gastric mucosa and alleviates symptoms such as epigastric pain and abdominal distension (Jiang et al. 2024). When combined with conventional therapies, these TCM interventions yield a synergistic effect that enhances overall treatment outcomes, particularly concerning *H. pylori* eradication and ulcer healing rates.

Moreover, the role of inflammatory factors in the development of peptic ulcers cannot be overstated. *H. pylori* infection triggers the release of inflammatory mediators, resulting in elevated serum levels of procalcitonin (PCT) and C-reactive protein (CRP) (Oktay et al. 2015; Manu et al. 2021). Prostaglandin E2 (PGE2) plays a critical role in gastric mucosal regeneration and ulcer healing (Konturek et al. 2005). Osteopontin (OPN) forms an immune barrier with gastric and duodenal epithelial cells, thereby impeding *H. pylori* invasion. However, *H. pylori* infection can enhance OPN secretion, leading to increased β-catenin levels and an immune response involving helper T lymphocytes (Pirzadeh et al. 2022; Mansour et al. 2020). Our results indicate that after 4 weeks of treatment, both groups exhibited reductions in serum PCT and CRP levels and increases in serum PGE2 levels compared to baseline. Notably, the observation group demonstrated significantly improved serum PCT, PGE2, and CRP levels, alongside lower serum OPN and β-catenin levels, suggesting that the combined therapy substantially mitigates inflammatory responses.

In our study, the incidence of adverse reactions was 18% in the control group compared to only 4% in the observation group. The presence of *Pinellia ternata*, known for its anti-emetic properties, along with *ginseng*, which alleviates dizziness and fatigue, contributed to a favourable safety profile. Anweiyang Capsule, as a proprietary Chinese medicine, not only avoids adverse reactions but also supports gastrointestinal function. However, a limitation of the study is that the sample size is small, and a larger sample size from multiple centers is needed to further validate these findings.

In conclusion, the combined application of Anweiyang Capsule and *Pinellia* Heart-Draining Decoction, alongside Western medicine quadruple therapy, demonstrates significant efficacy in managing peptic ulcers. This approach effectively enhances *H. pylori* eradication, promotes ulcer healing, and ameliorates inflammatory responses, establishing it as a valuable treatment strategy for patients with peptic ulcers. Further research is needed to elucidate the long-term benefits and safety of this integrative therapeutic approach.

## Data Availability

The datasets generated and analysed during the current study are available from the corresponding author on reasonable request.

## Abbreviations

TCM: Traditional Chinese Medicine
PCT: procalcitonin
CRP: C-reactive protein
PGE2: Prostaglandin E2
ELISA: enzyme-linked immunosorbent assay
